# DUOX2, a New Biomarker for Disseminated Gastric Cancer’s Response to Low Dose Radiation in Mice

**DOI:** 10.3390/cancers13164186

**Published:** 2021-08-20

**Authors:** Palak R. Parekh, Eduardo Solano-Gonzalez, Mariana B. Martins, Xinrong Ma, Kayla Tighe, Andrea Casildo, Andrew Zodda, Christopher Johnstone, Yannick Poirier, Javed Mahmood, Kavita Bhalla, Sheri Li, Rena G. Lapidus, France Carrier

**Affiliations:** 1 Veterans Affairs Maryland Health Care System, Baltimore, MD 21201, USA; palakparekh8579@gmail.com (P.R.P.); egonzalez@som.umaryland.edu (E.S.-G.); jmahmood@som.umaryland.edu (J.M.); kbhalla@som.umaryland.edu (K.B.); 2 Division of Translational Radiation Sciences, Department of Radiation Oncology, School of Medicine, University of Maryland, Baltimore, MD 21201, USA; MBMartins@som.umaryland.edu (M.B.M.); AZodda@som.umaryland.edu (A.Z.); Christopher.Johnstone@rmp.uhn.ca (C.J.); ypoirier@som.umaryland.edu (Y.P.); Sheri.Li@som.umaryland.edu (S.L.); 3 Program in Oncology, University of Maryland Marlene and Stewart Greenebaum Comprehensive Cancer Center, Baltimore, MD 21201, USA; 4 Translational Laboratory Shared Services, University of Maryland Marlene and Stewart Greenebaum Comprehensive Cancer Center, Baltimore, MD 21201, USA; XMa@som.umaryland.edu (X.M.); KaylaTighe@som.umaryland.edu (K.T.); ACasildo@som.umaryland.edu (A.C.); rlapidus@som.umaryland.edu (R.G.L.); 5 Department of Medicine, School of Medicine, University of Maryland, Baltimore, MD 21201, USA

**Keywords:** gastric cancer, low dose radiation therapy, Dual oxidase 2

## Abstract

**Simple Summary:**

The symptoms of early stomach cancer are often unremarkable, exhibiting only slight upper abdominal discomfort. By the time the symptoms become more obvious, the disease has usually progressed to an advanced stage resulting in more than 90% of inpatients presenting with locally advanced or metastatic cancer at the time of initial diagnosis. Disease that has spread into the abdomen is present in 10 to 30% of patients at the time of their initial surgery and is a frequent finding in patients who develop recurrent cancer. Treatment options are rather limited for these patients. Here, we designed a mouse model to evaluate the effect of very low dose of radiation to sensitize stomach cancer cells to conventional chemotherapy. Our data indicate that expression of DUOX2, an enzyme involved in the production of hydrogen peroxide, increases the odds of preventing cancer dissemination in response to low dose radiation and conventional chemotherapy.

**Abstract:**

Treatment options are rather limited for gastrointestinal cancer patients whose disease has disseminated into the intra-abdominal cavity. Here, we designed pre-clinical studies to evaluate the potential application of chemopotentiation by Low Dose Fractionated Radiation Therapy (LDFRT) for disseminated gastric cancer and evaluate the role of a likely biomarker, Dual Oxidase 2 (DUOX2). Nude mice were injected orthotopically with human gastric cancer cells expressing endogenous or reduced levels of DUOX2 and randomly assigned to four treatment groups: 1; vehicle alone, 2; modified regimen of docetaxel, cisplatin and 5′-fluorouracil (mDCF) for three consecutive days, 3; Low Dose- Whole Abdomen Radiation Therapy (LD-WART) (5 fractions of 0.15 Gy in three days), 4; mDCF and LD-WART. The combined regimen increased the odds of preventing cancer dissemination (mDCF + LD-WART OR = 4.16; 80% CI = 1.0, 17.29) in the DUOX2 positive tumors, while tumors expressing lower DUOX2 levels were more responsive to mDCF alone with no added benefit from LD-WART. The molecular mechanisms underlying DUOX2 effects in response to the combined regimen include NF-κB upregulation. These data are particularly important since our study indicates that about 33% of human stomach adenocarcinoma do not express DUOX2. DUOX2 thus seems a likely biomarker for potential clinical application of chemopotentiation by LD-WART.

## 1. Introduction

The prognosis for gastric cancer patients with intra-abdominal disseminated disease still remains very poor, with a median survival between 3 and 4 months without treatment [1]. Treatment options for disseminated gastric cancer may include systemic or hyperthermic intraperitoneal chemotherapy, local radiation, and now more targeted therapies such as immune checkpoint and human epidermal growth factor receptor 2 (HER2) antibodies [2,3,4]. Although patients initially respond to these therapies, the overall long-term outcome remains bleak, with less than 35% overall 5-year survival and only 2% once the disease metastasizes to the peritoneal cavity [5,6,7]. Recently, targeted therapies were approved by the US Food and Drug Administration as a first-line treatment for patients with locally advanced unresectable or metastatic HER2 positive gastric or gastroesophageal junction (GEJ) adenocarcinoma. Although promising, some of these new therapies have also been associated with increased treatment-related adverse events leading to treatment discontinuation [8,9].

Because gastrointestinal (GI) carcinomas are radiosensitive tumors, Whole Abdominal Radiation Therapy (WART) has been used in a palliative or adjuvant setting in an attempt to decrease intra-abdominal recurrence of disease [10]. Unfortunately, treatment of the entire abdomen with conventional 1.5–2.0 Gy per fraction inevitably limits a total dose of radiotherapy to be delivered and confines WART to adjuvant settings. In addition, the added toxicity limits the ability to combine this approach with full-dose chemotherapy, which is detrimental to the goal of treating disseminated disease. The objective of this study is to investigate the potential therapeutic application of chemopotentiation by Low Dose Fractionated Radiation Therapy (LDFRT) in a pre-clinical mouse model of disseminated intra-abdominal stomach cancer.

There is substantial pre-clinical and clinical evidence demonstrating the enhanced efficacy of chemotherapy when combined with LDFRT [11]. This approach has been particularly encouraging in head and neck cancer patients where the addition of LDFRT to an induction chemotherapy regimen of Carboplatin-Taxol was associated with a response rate of 86%. This was compared to previously reported response rates of ~55% for the same regimen without the use of LDFRT [12]. This paradigm has also been confirmed for safety and efficacy in a number of abdominal diseases, including pancreatic, ovarian, and endometrial cancer [13,14,15]. In the setting of a disease that requires both local controls as well as systemic dosing of chemotherapy, this offers the benefits of effective combined modality treatment that was hitherto considered not deliverable without additional toxicity (such as in WART). Nonetheless, clinical success with this approach has been variable, probably due in part to the lack of clear biomarkers to guide patient’ stratification. In the case of head and neck cancer, the status of p16 has been described as an important predictor of chemopotentiation by LDFRT [16], but this does not seem to be specific to LDFRT since p16 is also a predictor of the standard of care response [17]. In an effort to identify potential biomarkers for stomach cancer cells’ response to chemopotentiation by LDFRT, we recently identified DUOX2, an enzyme involved in hydrogen peroxide (H_2_O_2_) production, as an important mediator of hyper-radiosensitivity (HRS) and contributor to chemopotentiation by LDFRT in these cells [18]. In fact, the combined regimen of LDFRT and chemotherapy upregulated Reactive Oxygen Species (ROS) by more than 3 fold, and DUOX2 down-regulation abolished HRS [18]. ROS, including H_2_O_2_, can either promote cell survival or cell death depending on their intracellular concentration and localization [19]. Cancer cells, due to their enhanced metabolic activity, have constitutively higher ROS levels as compared to normal cells, and although high ROS levels are generally detrimental, cancer cells survive by upregulating prosurvival mechanisms and altering their antioxidant systems [20]. This implies that subtle bursts of ROS could reach toxic levels beyond the cellular antioxidative capacity in cancer cells, while a similar burst would be well tolerated in normal cells [20]. Here, we investigated the expression of DUOX2 in human tumors and designed pre-clinical animal studies to determine whether DUOX2 could be used as a biomarker of chemopotentiation by LDFRT for disseminated stomach cancer.

## 2. Materials and Methods

### 2.1. Cells and Cell Treatments

Human gastric cancer cell line NCI-N87 was obtained from American Type Culture Collection (Manassas, VA, USA) and grown as described in [18]. Normal human gastric epithelial cells GES-1 were obtained from Dr. Dawit Kidane-Mulat (University of Texas) and grown in RPMI 10% FBS [21]. Down-regulation of DUOX2 was performed by lentivirus transfection (Mission lentivirus packaging mix, Cat #SHP 001, Sigma Aldrich, St-Louis, MO, USA) according to the manufacturer’ s recommendations. pGFP-C-shDUOX2 lenti (TL313350) or pGFP-CSh scrambled lenti (TR-30021) (OriGene, Rockville, MD, USA) and packaging plasmids were used. Detection of GFP expression and Western blots were performed to determine infection efficiency. Chemotherapy and cells irradiation were performed as described in [18], except that irradiation was performed twice a day with 6 h intervals between the fractions. The primary mouse gastric cancer cell line NCC-S1 (S1) and its derivative NCC-S1M (S1M) were obtained from Hark Kyun Kim, National cancer center, Republic of Korea. The S1 cells were established from a Villin-cre; Smad4 (F/F); Trp53 (F/F); Cdh1 (F/wt) mouse, and its metastatic variant cell line NCC-S1M (S1M) exhibiting CSC-like features was isolated from lung metastasis that developed from heterotopic allografts of NCC-S1 [22,23]. The mouse gastric cancer cells were maintained and grown in RPMI, 10% FBS. Western blots were performed as described in [18] with the exception that DUOX2 proteins were extracted with the Membrane Protein Extraction Kit Mem-PER^TM^ Plus according to the manufacturer’s recommendations (Cat no: 89842, Thermo Scientific, Waltham, MA, USA). For Stat1 expression, nuclear protein extracts were used, the blots were hybridized with Stat1 rabbit polyclonal antibody (Cat no: 9172, Cell Signaling Technology, Danvers, MA, USA) stripped and rehybridized with phospho specific Stat1 antibodies (Cell Signaling Technology, Phospho-Stat1 (Ser727) #9177; Cell Signaling Technology, Phospho-Stat1 (Tyr701) #9167)).

Cells irradiation, clonogenic survival assays, and DUOX2 mRNA analysis were performed as described in [18]. In order to evaluate the chemopotentiation effect of LDFRT, chemotherapy was used at IC_50_ [18].

### 2.2. Protein Carbonyl 

Serum protein oxidation from tissue culture media was measured by an OxiSelect™ Protein Carbonyl ELISA Kit (Cat no STA-310, Cell Biolabs, San Diego, CA, USA), and serum protein oxidation from mouse serum (10 μg) was performed with a fluorometric Protein carbonyl content assay kit (Cat no ab235631, Abcam, Cambridge, MA, USA) according to the manufacturers’ recommendations. Data were expressed either as a percentage of the respective untreated samples (scrambled RNA or siRNA DUOX2) set at 100% or as μM serum protein carbonyl content.

### 2.3. Mouse Angiogenesis Profiler Array

The angiogenesis proteome profiler was conducted according to the manufacturer’s recommendations using the Mouse Angiogenesis Array Kit (cat # ARY015 R&D systems, Minneapolis, MN, USA). Briefly, the tumors were separated and transferred to 1 ml protease inhibitor cocktail containing PBS (Sigma-Aldrich Co., St-Louis, MO, USA) and Triton-X-100. Three to four tumors from each treatment group were then pooled and disrupted using TissueRuptor II (Qiagen, Valencia, CA, USA) at 100% amplitude with 30 s cycle pulse. Tissue lysates were then centrifuged at 4 °C at 10,000× *g* for 5 min. The supernatant was collected, and the amount of protein in each sample was estimated using the BCA protein assay kit (cat # 23225, Thermo Scientific), and saved immediately at −80 °C. A total of 300 µg of tissue lysates from each group was mixed with a cocktail of biotinylated detection antibodies, added into each well containing antibodies pre-coated membranes, and incubated overnight at 4 °C. The membranes were then washed and detected by chemiluminescence following a 30 min incubation with Streptavidin-HRP antibody. A total of 1 mL of the detection buffer was pipetted onto the membrane. Quantification of pixel densities in the developed X-ray film was performed with Image-J. The background was subtracted from each pair duplicate, and the average was plotted as relative fold change compared to vehicles in respective groups. All data were presented as the means ± SD. Statistical analyses were performed by one-way analysis of variance (ANOVA) using GraphPad Prism 6.0 software. *p* < 0.05 was considered significant.

### 2.4. TCGA Stomach Adenocarcinoma

Data from a TCGA’s data set (449 stomach adenocarcinoma (STAD) samples were provided by the UALCAN web resource for analyzing cancer OMICS data (http://ualcan.path.uab.edu/index.html [24], accessed on 28 July 2021). Significance of difference estimated by Student’s *t*-test considering unequal variance.

### 2.5. Animal Studies

All animal procedures were performed with the assistance of the University of Maryland Marlene and Stewart Greenebaum Comprehensive Cancer Center Translational Laboratory Shared Services, reviewed and approved by an Institutional Animal Care and Use Committee (Protocol # 0317002) at the University of Maryland Medical School. Orthotopic model: In a previous report, 50 × 10^6^ NCI-N87-GFP cells were injected I.P. to detect cancer cells progression by fluorescence imaging [25]. Here, we determined in a pilot study (Figure S4) that approximately 30 × 10^6^ cells were sufficient to achieve efficient tumor engraftment in all animals and allow monitoring of tumor progression by fluorescence imaging (Xenogen IVIS) in real-time in live animals. We, therefore, injected 28 × 10^6^ cells I.P. in each animal. Because these cells have been reported to grow well in the abdominal cavity of female athymic nu/nu mice [25] we only used female mice that were injected I.P. with metastatic stomach carcinoma NCI-N87 (5822) cells stably transfected with either scrambled-GFP or DUOX2 shRNA-GFP plasmid (OriGENE, Rockville, MD, USA) in 400 μL PBS [18]. Once tumors had formed (14 days), the mice were randomized based on similar fluorescence intensity into 4 treatment groups of 7 to 8 mice each. The assumption used for sample size was based on an earlier study using a similar fluorescence detection method and NCI-N87-GFP cells, showing significant differences between control and treated groups with 5 animals per group [25]. The mice were treated for 3 consecutive days as follows; Group1 untreated (vehicle alone), Group 2 mDCF alone, Group 3 Low Dose Fractionated Whole Abdomen Radiation Therapy (LD-WART) alone and Group 4 LD-WART plus mDCF. In order to evaluate the chemopotentiation effect of LD-WART, the mDCF in gastric cancer Xenograft model was used at 50% of the Maximum Tolerated Dose (MTD) in nude mice [26]. Mice treated with mDCF received 8.5 mg/kg 5-fluorouracil (5-FU) and 10 mg/kg DTX (docetaxel) on day 1, 8.5 mg/kg 5-FU on day 2 and 8.5 mg/kg 5-FU and 3 mg/kg Cisplatin on day 3 (Figure S1). All treatments were well tolerated and did not result into any significant toxicity in any treatment groups as evidenced by no body weight loss during the study (Figure S2). Of all treatment groups, a single mouse died 14 days following treatment initiation. The mouse was from the scrambled RNA group that received chemotherapy alone.

### 2.6. Animal Irradiation

#### LD-WART

Once anesthetized using isoflurane the mice were placed in a prone position on an animal platform and secured in place. Animals received five 0.15 Gy fractions to the whole abdomen in 3 consecutive days as follows: day one, a single 0.15 Gy fraction, day 2 and 3 b.i.d. fractions 4–6 h apart [12]. The irradiations were performed using the Small Animal Radiation Research Platform (SARRP, Xstrahl, Atlanta, GA, USA) operating at 220 kVp, 0.15 mm Cu filtration, and 0.5 mA. The unit was calibrated with a NIST-traceable PTW TN30013 farmer-type ionization chamber and a PTW Unidose electrometer (PTW, Breisgau, Germany) following the American Association of Physicists in Medicine (AAPM) Task Group 61 (TG-61) calibration protocol for X-ray irradiators [27]. The tube current was decreased from the typical 13 mA to produce a lower dose rate of ~0.1 Gy/min and irradiation times of ~40 s per field to reduce errors due to time rounding. Mice were irradiated sequentially, placed in groups of three on the SARRP’s robotic stage. First, the animals were placed at the isocenter using the SARRP’s rotation stage and the positioning lasers aligned to the hips. Then, irradiations were performed bilaterally using an anterior-posterior/posterior-anterior (AP/PA) geometry with a custom 4 cm collimator at a source-to-axis distance of 35 cm. The stage was translated from one animal to the next for one field, then the gantry was rotated, and the other fields delivered. Irradiation times were calculated on pre-scanned representative animals using the SARRP’s treatment planning system, Muriplan, and were verified using a mouse-mimicking plastic phantom and a pinpoint ionization chamber (IBA, Louvain-La-Neuve, Belgium) cross-calibrated with the NIST-traceable farmer chamber. For each radiation delivery, the SARRP’s portal camera was used to take a digital portal image of the irradiated area to verify field placement. Portal images were acquired of each irradiated mouse to define the radiation field set to the top of the diaphragm in full exhale and the obturator foramen, as described previously in a clinical protocol [10]. The diaphragm was clearly visible during treatment as the lungs came in and out of the treatment field with breathing.

### 2.7. Electrophoretic Mobility Shift Assay (EMSA)

EMSA was performed with a LightShift Chemiluminescent kit according to the manufacturer’s recommendations (Cat no: 20148, Thermo Scientific, Waltham, MA, USA). Briefly, NCI-N87 cells were treated as described in [18] with the exception that irradiation was performed b.i.d. Nuclear proteins were extracted and 1.6 μg was used with 40 fmole of 5′ biotinylated DNA probe (IDT, Coraville, IA, USA). The DNA probe was a double stranded 40 nucleotides sequence from the human DUOX2 promoter containing one NF-κB binding site:

Biotin-5′-GATGGCAGCGGTGCAGGGGAATTCCCCGGGGGAGAAGCGG-3′. Where indicated 0.5 μg of p-p65-NF-κB (Ser536) monoclonal rabbit antibody (Cat no 3033; Cell Signaling, Danvers, MA, USA) was used.

### 2.8. RT-PCR

DUOX2 expression was measured as described before [18]. Briefly, RNA was isolated by RNeasy Mini kit (Qiagen, Valencia, CA, USA) and cDNA synthesized with 1 μg of RNA (human DUOX2) or 500 ng RNA (mouse DUOX2) using high-capacity cDNA kit (Applied Biosystems, USA Ref No 4368814). Real-Time PCR was performed in a total volume of 20 μL using 1 μL of the first strand cDNA synthesis mixture as a template, 0.4 μM forward and reverse primer and 10 μL of 2X SYBRGreen PCR Master Mix (Applied Biosystems, USA Ref No: A46012). Triplicate reactions were carried out in MicroAmp optical 96 well reaction plate (Applied Biosystems Ref No: 8010560), on a 7300 Real-Time PCR system (Applied Biosystems^®^, Carlsbad, CA, USA), and fluorescence was quantified against standards. The universal thermal cycling parameters were used as recommended (10 min, activation at 95 °C, followed by 40 cycles of 15 s at 95 °C and 1 min at 60 °C). The data for all genes were standardized against the *Gapdh* data. Control wells containing SYBER Green PCR master mix and primers without sample cDNA were used for background determination. Proprietary Qiagen primers were used: Human DUOX2 (Qiagen Catalog no 330001, PPH09835F: Primers sequence: UniGene no: Hs.71377, RefSeq Accession no: NM_014080.4, Reference position: 4305), Human GAPDH (Qiagen Catalog no 330001, PPH00150F: Primers sequence: UniGene no: Hs.592355, RefSeq Accession no: NM_002046.5, Reference position: 842), Mouse DUOX2 (Qiagen Catalog no 330001, PPM40846A:Primers sequence: UniGene no: Mm.358846, RefSeq Accession no: NM_177610.2, Reference position: 2505), Mouse GAPDH (Qiagen Catalog no 330001, PPM02946E, Entrez Gene Id: 14433, RefSeq Accession no: NM_008084, Reference Position: 478).

### 2.9. Immuno Histo Chemistry

Human stomach adenocarcinoma tissue arrays (37 samples) with progressive disease were obtained from Pantomics, Inc. (Cat no, STC 962, Richmond, CA, USA) and analyzed by immunohistochemistry for DUOX2 (mouse monoclonal MIL-MABN787, 1/300, Millipore Sigma, St-Louis, MO, USA), and macrophages (CD68, mouse monoclonal EBS-14-0688-82, 1/1000). All samples were collected from treatment naïve patients. Patients’ characteristics are provided in Supplemental Tables S1 and S2. Staining was scored blinded by Dr. Langxing Pan, a pathologist at Pantomics, Inc. DAKO pan-cytokeratin antibody kit and normal mouse serum were used as positive and negative controls. Dr. Pan’ scoring criteria were: “0” = negative, “0.5” = negative with some weak but suspicious staining; “1” = weak staining; “2” = moderate staining; “3” = strong staining. For this analysis, Staining ≥ 2.8 was considered strong DUOX2 positive, score of “0” was considered DUOX2 negative. Scoring data are provided in Supplemental Tables S1 and S2.

### 2.10. Statistics

Statistical analyses were performed on the relative levels of protein carbonyl, surviving fractions of mouse gastric cancer cells, RT-PCR, intensity of immune cells staining, mouse angiogenesis proteome data and mobility shift assays. Experiments were performed 2 to 3 times, depending on the assay and were analyzed by either Student’s *t* test (1-tailed) with an Excel spreadsheet or 1 way ANOVA with Dunnett’s multiple comparison test (GraphPad Prism6 and 9). Probability values < 0.05 were considered significant. Odds ratios were calculated on fluorescence of disseminated human gastric cancer cells in mouse abdomen with GraphPad Prism9 with confidence interval sets at 80%.

## 3. Results

### 3.1. Upregulation of DUOX2 and Protein Oxidation In Vitro

**Figure 1:**
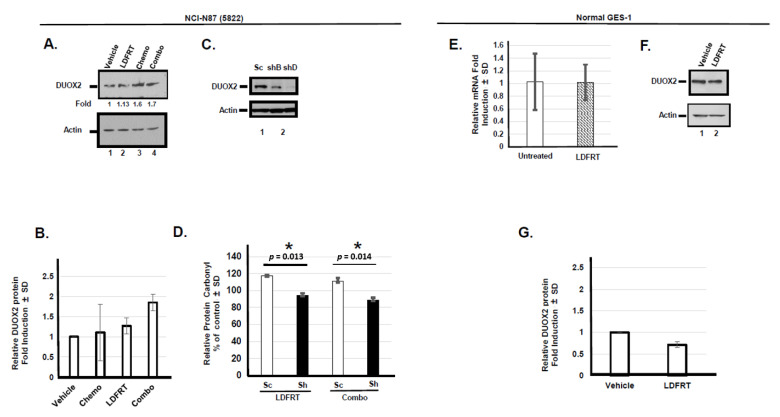
DUOX2 expression and activity in human gastric cells. (**A**) Western blots analysis of gastric cancer NCI-N87 (5822) cells exposed to the indicated treatment. (**B**) Quantitation of the Western blots. Fold induction was measured by densitometry and normalized to Actin and the untreated sample (Vehicle). (**C**) Down-regulation of DUOX2 in NCI-N87 cells, shD cells were used for all other shDUOX2 experiments. (**D**) Relative protein carbonyl content in the media of NCI-N87 cells expressing endogenous (Sc: Scrambled) or reduced (Sh) DUOX2 levels expressed as a percentage of the respective untreated samples, set at 100%, in the indicated cells exposed to LDFRT or the combined regimen of mDCF and LDFRT. * *p* values, Student’s *t* test. *p* < 0.05 is considered significant. (**E**) DUOX2 mRNA expression in normal gastric epithelial GES-1 cells untreated or exposed to radiation (LDFRT). *n* = 3. (**F**) Western blots of DUOX2 in GES-1 cells exposed to the indicated treatment. (**G**) Quantitation by densitometry of the GES-1 Western blots. *n* = 3.

In order to evaluate the role of DUOX2 in chemopotentiation by LDFRT in a pre-clinical model, we first confirmed that LDFRT and the combined regimen resulted in DUOX2 upregulation in human stomach cancer cells. Since several clinical trials have established that combination chemotherapy increases overall survival as compared to single-agent therapy in advanced metastatic gastric cancer [28], we modeled our pre-clinical study on the current approaches. The data are shown in Figure 1A,B indicate that as we reported before in two human stomach cancer cell lines [18], three consecutive days of LDFRT (0.15 Gy) or a combination of a modified regimen of docetaxel, cisplatin, and 5-fluorouracil (mDCF) and LDFRT resulted in DUOX2 protein upregulation in the NCI-N87 stomach cancer cells. Since we previously showed that these treatments resulted into increased intracellular Reactive Oxygen Species (ROS) in these cells [15], we then measured the levels of serum protein carbonyl content as a readout of DUOX2 activity in the media of cells expressing endogenous or reduced DUOX2 levels (Figure 1C,D). Our data indicate that the relative levels of serum protein oxidation were significantly decreased following LDFRT or the combined regimen (*p* = 0.013 and 0.014 respectively) in the media of cells expressing lower DUOX2 levels suggesting that indeed this assay was a good readout of DUOX2 activity. These data are also in good agreement with earlier studies demonstrating that down-regulation of DUOX2 reduces ROS levels in cells [29,30]. We then verified that LDFRT did not upregulate DUOX2 mRNA or protein levels in normal epithelial gastric cells (Figure 1E–G), confirming our previous report with normal small intestinal epithelial cells [18].

#### 3.1.1. Role of DUOX2 in Disseminated Gastric Cancer Response to mDCF and LD-WART

**Figure 2:**
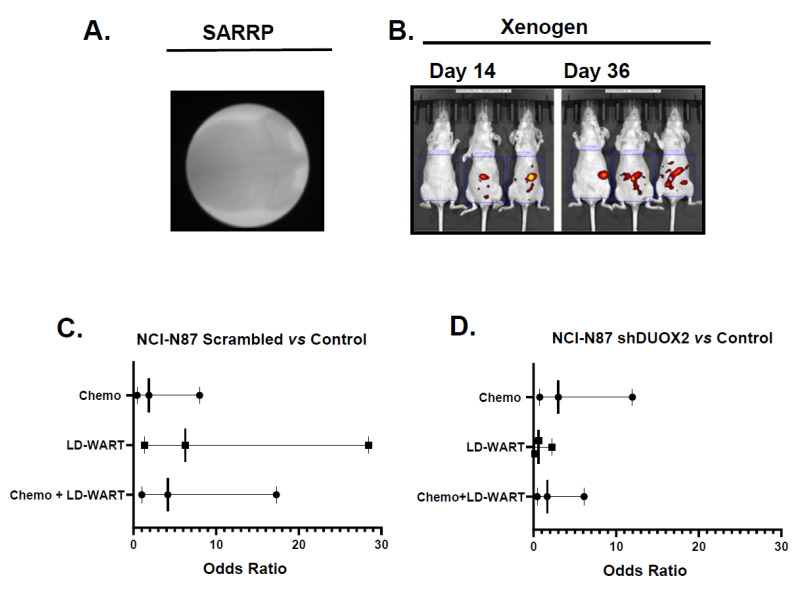
(**A**) Representative image obtained with a SARRP’s portal camera to verify field placement of irradiated field (whole mouse abdomen). (**B**) Representative fluorescence image (closed abdomen) obtained with a Xenogen IVIS optical imager on day 14 and 36 post-injection of human gastric cancer cells stably transfected with pGFP-C-shDUOX2 lenti. (**C**,**D**) Odds of reducing cancer dissemination as measured by fluorescence intensity being less than 25% of the intensity of day 1 at day 45. (**C**) Odds of tumor expressing endogenous DUOX2 levels treated with the indicated treatment compared to untreated animals. (**D**) Odds of tumor expressing reduced DUOX2 levels treated with the indicated treatment compared to untreated animals. *n* = 7–8.

In an effort to develop a pre-clinical model to evaluate the potential application of chemopotentiation by Low Dose Fractionated Radiation Therapy (LDFRT) for disseminated gastric cancer, the human stomach cancer NCI-N87 cells expressing endogenous or reduced DUOX2 levels (Figure 1C) were then injected orthotopically in the abdomen of mice and treated, as described in the Material and Methods. In order to measure the effect of the treatments on cancer dissemination in the abdomen we measured fluorescence intensity of the whole abdomen at different times following treatments (Figure 2A,B). Our data indicate that the abdominal fluorescence intensity of the majority of mice (5/7) in the control untreated group at day 45 was at least 25% higher than the intensity of day 1. We thus used this parameter to calculate the odds that a treatment would result in fluorescence intensity being less than 25% of the intensity of day 1 at day 45 as an indication of treatment efficiency on cancer progression and dissemination. Data shown in Figure 2C indicate that the odds ratio (OR) of preventing cancer dissemination as measured by fluorescence intensity being less than 25% of day 1 at day 45 were higher in all treated DUOX2 positive tumor compared to untreated tumor (chemo OR = 1.87; 80% CI = 0.44, 8.01; LD-WART OR = 6.25; 80% CI = 1.37, 28.47; chemo + LD-WART OR = 4.16; 80% CI = 1.0, 17.29) with LD-WART being the most efficient. On the other hand, chemotherapy alone was the most efficient in the tumor expressing reduced DUOX2 levels (Figure 2D, chemo OR = 3.0; 80% CI = 0.75, 11.98; LD-WART OR = 0.6; 80% CI = 0.16, 2.21; chemo + LD-WART OR = 1.66; 80% CI = 0.45, 6.13) with no added benefit from LD-WART when compared to untreated or chemotherapy alone. Due to the small number of animals used (7–8) the confidence intervals (CI) were calculated at 80%. Nonetheless, the CI did not span 1.0 for tumor expressing endogenous DUOX2 levels exposed to LD-WART alone or combined with chemotherapy when compared to the untreated control. On the other hand, the CI did span 1.0 for all tumor expressing reduced DUOX2 when compared to untreated samples. The tendencies for the DUOX2 positive tumors are thus considered reasonable and support a role for DUOX2 in mediating radiosensitivity to gastric cancer tumors.

**Figure 3:**
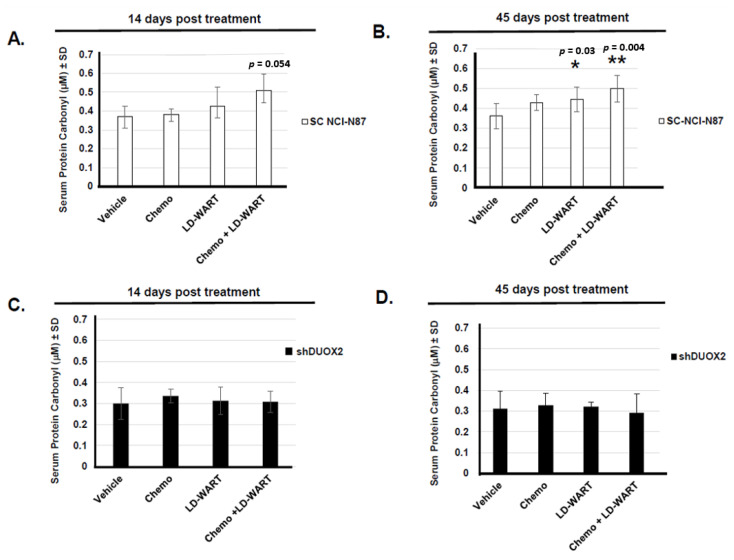
DUOX2 activity in vivo. (**A**,**B**) Serum protein carbonyl content from serum of mice bearing tumors expressing endogenous (scrambled, white boxes) DUOX2 levels at 14 (**A**) or 45 (**B**) days post end of the indicated treatments (*n* = 7–8 mice). (**C**,**D**) Same as (**A**), (**B**) except that serum was obtained from mice bearing tumors expressing reduced DUOX2 levels (shDUOX2, black boxes). *n*= 6–8 mice. Data are compared to serum from untreated (Vehicle) mice. Data were evaluated by 1 way ANOVA with Dunnett’s multiple comparison test. * *p* < 0.05, ** *p* < 0.005. *p* < 0.05 is considered significant.

In an effort to investigate the role of DUOX2 in preventing cancer dissemination in vivo, we next measured serum protein carbonyl content as a read out of DUOX2 activity in mice bearing stomach tumors expressing endogenous or reduced DUOX2 levels following the different treatments. Our data (Figure 3A) indicate that the level of protein carbonyl increase in the serum of mice bearing DUOX2 positive tumor following the combined regiment of chemotherapy and LD-WART as early as 14 days post-treatment but only reached significance 45 days post-treatment (Figure 3B). Exposure to LD-WART also resulted in significant protein carbonyl elevation 45 days post-treatment while no significant increase was observed with any treatment up to 45 days post-treatment in the serum of mice expressing reduced DUOX2 levels (Figure 3C,D, shDUOX2). These data are not only in good agreement with our previous cell-based data (Figure 1D, [18]) but also support a role for DUOX2 in sensitizing gastric cancer cells to low dose radiation in vivo.

#### 3.1.2. Effect of LD-WART on Angiogenesis

**Figure 4:**
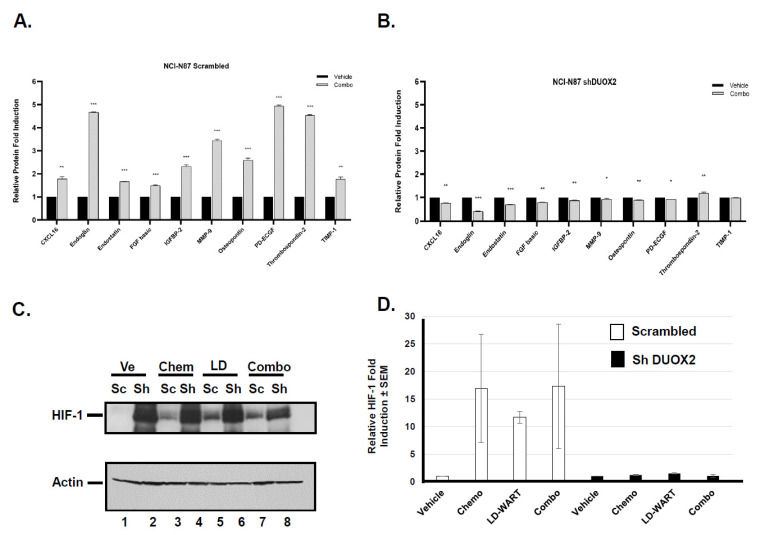
Effect of combined regimen of mDCF and LD-WART on angiogenesis and HIF-1 in vivo. (**A**) Mouse angiogenesis profiler array performed on tumors expressing endogenous DUOX2 levels. Three to four tumors of each treatment groups were pooled and analyzed as described in Material and Methods. Data are presented as fold induction of respective control (Vehicle) after background subtraction ± SD. (**B**) Same as (**A**) except that proteins were extracted from DUOX2 negative tumors (shDUOX2). Statistical analyses were performed by one-way analysis of variance (ANOVA) using GraphPad Prism 6.0 software. * *p* < 0.05, ** *p* < 0.01, *** *p* < 0.001. *p* < 0.05 was considered significant. (**C**) Western blot of HIF-1 protein extracted from tumors expressing endogenous (Sc = scrambled) or reduced (Sh-shDUOX2) levels. Actin was used as loading control. Ve = Vehicle, Chem = chemotherapy (mDCF), LD = LD-WART, Combo = mDCF + LD-WART. (**D**) Quantitation of HIF-1 Western blots expressed as fold induction after normalization to Actin and respective untreated (Vehicle) control.

Ionizing radiation and particularly low dose radiation have been shown to have angiogenic potential [31,32]. Because an increase in angiogenesis could sensitize cancer cells to radiation through tissue reoxygenation [33,34], we then measured the effect of our regimen on angiogenesis factors in tumor expressing endogenous or reduced DUOX2 levels. We performed mouse angiogenesis profiler arrays to simultaneously evaluate the expression of 53 different angiogenic factors. The data shown in Figure 4A indicate that at least 10 angiogenic factors were significantly upregulated in DUOX2 positive but not in DUOX2 negative tumors (Figure 4B) following exposure to our combined regimen of mDCF and LD-WART. We also measured the response of the angiogenic factors to single modality (mDCF and LD-WART) and found similar results with no induction in the DUOX2 negative tumors (Figure S4). Low dose radiation has also been shown to transiently upregulate VEGF seven to twenty-eight days following total mouse body irradiation [32]. Here, we harvested the tumors forty-five days after treatments initiation, which may explain why we did not observe VEGF up-regulation in any treatment groups (Supplemental Figure S4). While most factors in this array are pro-angiogenic, three factors, Endostatin, Thrombospondin-2, and TIMP-1 are anti-angiogenic. It thus appears that the overall effects is pro-angiogenic since low dose radiation can promote endothelial cells proliferation and improve neovascularization [35]. Because solid tumors can also develop hypoxic regions that can contribute to radio and chemo resistance, we next measured the levels of the Hypoxic Inducible Factor (HIF-1) in the tumors expressing endogenous or reduced DUOX2 levels in response to our treatments. The data shown in Figure 4C indicate that although HIF-1 was upregulated by all treatments in the DUOX2 positive tumors, its basic level was much higher in the DUOX2 negative tumors (sh DUOX2, Figure 4C lane 2) and did not change in response to any treatments in these tumors (Figure 4C lanes 4, 6, 8, Figure 4D). The increased HIF-1 levels in the shDUOX2 tumors may thus have contributed to their insensitivity to LD-WART (Figure 2D).

### 3.2. Expression of DUOX2 in Human Stomach Cancer

**Figure 5:**
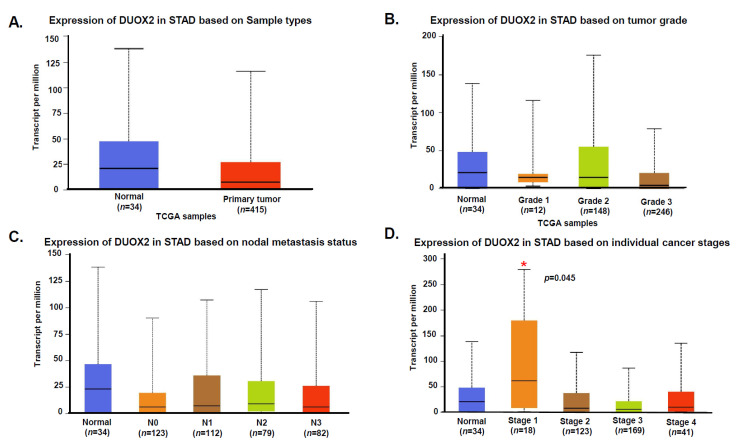
TCGA data of DUOX2 mRNA expression in human Stomach adenocarcinoma (STAD) tumors. (**A**) Levels of DUOX2 mRNA expression in 34 Normal and 415 Primary stomach adenocarcinoma. (**B**) Levels of DUOX2 mRNA expression based on tumor grade. The number of samples of each tumor grade are indicated in parenthesis. (**C**) Levels of DUOX2 mRNA expression based on nodal metastasis status. (**D**) Expression of DUOX2 mRNA based on individual cancer stages. Data provided by the UALCAN web resource for analyzing cancer OMICS data (http://ualcan.path.uab.edu/index.html [24], accessed on 28 July 2021). Significance of difference estimated by Student’s *t*-test considering unequal variance. * *p* < 0.05.

**Figure 6:**
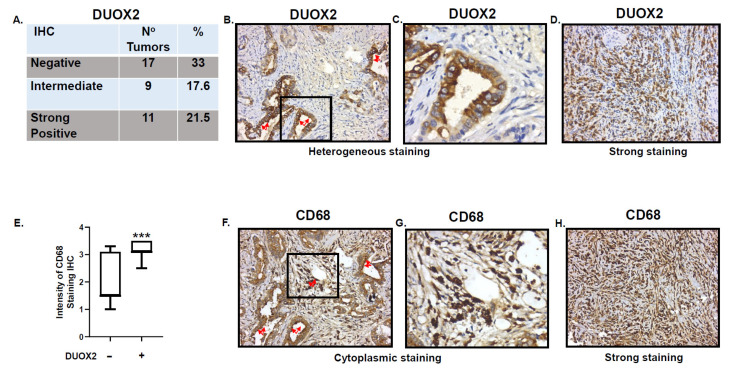
Immuno-Histo-Chemistry (IHC) in 37 human adenocarcinoma samples. (**A**) Staining scored blinded by a pathologist at Pantomics, Inc. Staining > 2.8 was considered strong DUOX2 positive, score of “0” was considered DUOX2 negative. Percentage of strong staining and negative DUOX2 staining are indicated. (**B**) Example of heterogeneous (positive and negative) DUOX2 staining in well differentiated/low grade gastric adenocarcinoma (Malignant 1). Red arrows indicate cells expressing DUOX2. (**C**) Expanded view of inset from (**B**). (**D**) Example of strong DUOX2 staining in poorly differentiated/high grade stomach adenocarcinoma. (**E**) Intensity of CD68 staining in DUOX2 negative and positive samples. Student’s *t*-test, *** *p* = 0.0005. (**F**) CD68, red arrows indicate macrophages tumor and stroma infiltration in same tissue as (**B**) expressing DUOX2. (**G**) Expanded view of inset from (**F**). (**H**) IHC in poorly differentiated/high grade stomach adenocarcinoma showing strong CD68 staining. Images dimensions are 450 × 383 pixels (W × H).

To evaluate the potential clinical significance of these data we first consulted the TCGA data base and the Human Protein ATLAS. Data from a TCGA’s data set (449 stomach adenocarcinoma (STAD) samples, Figure 5) provided by the UALCAN web resource for analyzing cancer OMICS data (http://ualcan.path.uab.edu/index.html [24], accessed on 28 July 2021) indicate that there are no DUOX2 mRNA statistical differences between normal and primary tumors based on sample types, tumor grades, or nodal metastasis status. Nonetheless, Stage 1 samples show a statistically significant (*p* = 045) increase of DUOX2 mRNA expression as compared to normal tissue. Accordingly, the Human Protein ATLAS indicates that expression of DUOX2 is not a prognostic factor in stomach cancer. To validate these data, we next performed immunohistochemistry (IHC) analysis of DUOX2 expression on a panel of human stomach samples from normal, premalignant and cancers tissues with progressive grades. Our data confirmed that DUOX2 protein levels did not correlate with tumor type, grade or stages (data not shown), however we observed that more than 30% of human stomach adenocarcinoma are actually negative for DUOX2 while 21% show strong staining (Figure 6A–D). The reason for the variability of DUOX2 expression in human stomach cancer is not known but may be based on the levels of inflammation. In fact, our data indicate that six of the seven (86%) gastritis samples we examined expressed strong levels of DUOX2 in the surface of epithelial cells (data not shown). Based on these data and the role of immune cells in inflammation we next stained the tissues with the macrophage marker CD68 to measure levels of infiltrating macrophages in gastric tumor negative or positive for DUOX2 expression. Our data indicate that expression of DUOX2 corresponds to a significant increase of macrophages infiltration (Figure 6E) and that macrophages are not only recruited in tissue expressing DUOX2 (Figure 6F) but also in the stroma surrounded by cells expressing DUOX2 (Figure 6F,G).

### 3.3. Regulation of DUOX2 Expression in Response to mDCF and LDFRT

**Figure 7:**
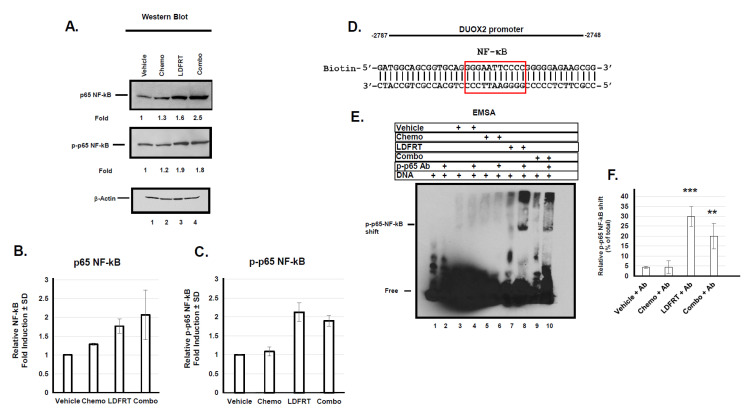
NF-κB response to mDCF and LDFRT in NCI-N87 cells. (**A**) Western blot analysis of total (p65) and Ser536 phopshorylated (p-p65) NF-κB. Fold induction of total (p65) and phospho NF-κB (p-p65) normalized to Actin and untreated (Vehicle) control. (**B**,**C**) Densitometry quantitation of p65 and phospho p65 Western blots. (**D**) Sequence of NF-κB binding site in human DUOX2 promoter. (**E**) EMSA of cells exposed to the indicated treatments. Human gastric cancer cells NCI-N87 were exposed to the indicated treatment, nuclear protein extracted, and incubated with the indicated components (+) and run on a native gel. Shifted protein complex is indicated. (**F**) Densitometry quantitation of antibody shifted p-p65 NF-κB band expressed as a percentage of total biotinylated DNA probe. Data were analyzed by 1 way ANOVA with Dunnett’s multiple comparison test. ** *p* < 0.005, *** *p* < 0.0005.

To better understand the molecular mechanisms underlying DUOX2 response to the combined regimen we next evaluated the role of transcription factors that could potentially regulate DUOX2 expression. The human DUOX2 proximal promoter only contains a handful of well characterized transcription factor binding sites including NF-κB, STAT1, STAT3, c-Fos, c-Jun, and C-EBPα. NF-κB and Stat1 are both upregulated in response to low dose radiation [36]. We thus evaluated their potential role in DUOX2 expression under the conditions used here. DUOX2 promoter contains 7 NF-κB binding sites and low dose radiation has been shown to increase NF−κB phosphorylation and DNA-binding activity [36,37]. The data shown in Figure 7A–C indicate that indeed LDFRT or the combined regimen of mDCF and LDFRT upregulated total and Ser536 phosphorylated NF-κB in human gastric cancer cells. Moreover, EMSA data indicate that phosphorylated NF-κB can bind to an NF-kB responsive element in the DUOX2 promoter (Figure 7D) but only when cells are exposed to LDFRT or the combined regimen (Figure 7E,F, lanes 8, 10). This is in good agreement with p-p65 NF-κB upregulation in response to these treatments (Figure 7A, lanes 3, 4) and supports a role for NF-κB in DUOX2 upregulation in response to LDFRT.

**Figure 8:**
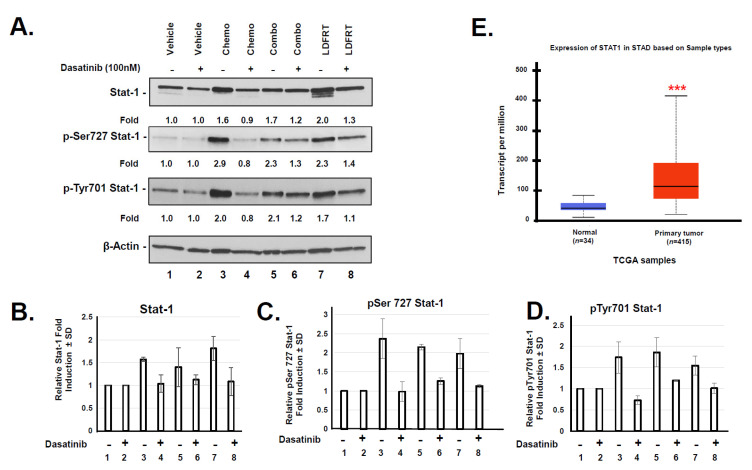
Stat-1 response to mDCF and LDFRT in NCI-N87 cells. (**A**) Western blot analysis of total Stat-1, and Stat-1 phosphorylated at Serine 727 (p-Ser727) and Tyrosine 701 (p-Tyr701). Cells were exposed to the indicated treatment and pre-treated (+) or not (−) with the Src kinase inhibitor Dasatinib. (**B**–**D**) Fold induction measured by densitometry and normalized to Actin and the untreated sample (Vehicle). (**E**) Expression of Stat1 in normal and stomach adenocarcinoma (STAD) from TCGA samples accessed on 28 July 2021. [24]. Student’s *t*-test, *** *p* < 0.0005.

As mentioned above, Stat-1 is another potential regulator of DUOX2 in response to LDFRT. The signal transducers and activators of transcription (STATs) can be activated by Src kinases in a variety of human malignancies [38]. Early studies have shown that Src kinase can be activated in response to doses of radiation as low as 0.05 Gy [39]. Maximal activation of transcription by Stat1 requires both tyrosine and serine phosphorylation [40,41]. Because up-regulation and activation of Stat1 are essential for DUOX2 expression [42], we investigated whether this signaling axis is involved in DUOX2 expression in response to LDFRT combined to chemotherapy. The data shown in Figure 8A–D indicate that indeed phosphorylated Stat1 is upregulated in response to mDCF, LDFRT, or the combined regimen. Inhibition of the Src kinases with Dasatinib prior exposing the cells to the indicated treatments reduced Stat-1 up regulation in response to mDCF, LDFRT or the combined regimen. These data are in good agreement with previous studies [26] and support a role for Stat-1 involvement in DUOX2 response to the proposed regimen. Moreover, an analysis of the TCGA stomach adenocarcinoma (STAD) data set (Figure 8E, [24]) indicate that Stat-1 is significantly upregulated in STAD compared to normal tissue. These data suggest that Stat1 could potentially be an additional biomarker for human gastric tumors response to LDFRT.

### 3.4. Potential Role of DUOX2 in Cancer Stem Cells Response to mDCF and LDFRT

**Figure 9:**
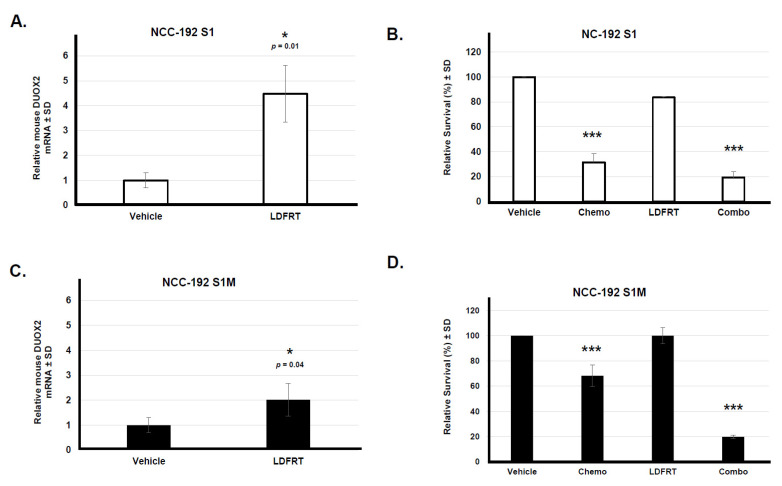
Efficacy of combined regimen in mouse gastric cancer cells. (**A**,**C**) Expression of DUOX2 mRNA in response to LDFRT in primary mouse gastric cancer cell line NCC-S1 (S1) and its derivative CSC-like NCC-S1M (S1M). Data are compared to their respective untreated samples. * *p* < 0.05, Student’s *t*-test. (**B**,**D**) Clonogenic survival assays were performed in triplicate on cells (NCC-S1, white boxes (**B**) and NCC-S1M, black boxes, (**D**)) treated with the indicated treatments. Relative survival is expressed as a percentage of the respective untreated (Vehicle) control. *** *p* < 0.0005, 1 way ANOVA with Dunnett’s multiple comparison test.

Because cancer stem cells (CSCs) are notoriously radio- and chemo-resistant and often responsible for cancer recurrence following treatments [43,44,45], cancer therapy strategies should aim at targeting not only the cancer cells, but also the CSCs. As proof of principle to determine whether the combined regimen of mDCF and LDFRT could affect CSCs, we treated primary mouse gastric cancer cells NCC-S1 (S1) and their CSC-like variant NCC-S1M (S1M) [23] with the same modalities used for the human gastric cancer cells (Figure 1, [18]). The S1M cells are a S1 metastatic variant exhibiting CSC-like characteristics such as increased tumorigenic potential, chemoresistance, and tumorspheres formation [23]. We first verified that DUOX2 could be upregulated in these cells. The data shown in Figure 9A indicate that DUOX2 could be significantly upregulated by LDFRT in both mouse cell lines (Figure 9A,C) as it was in human gastric cancer cells (Figure 1). Using the parent NCC-S1 cells as control, we compared the efficacy of the regimen to the CSC-Like subpopulation, NCC-S1M. Data shown in Figure 9B, D indicate that, as could be expected, the CSC-Like population were more resistant to mDCF (Chemo) and LDFRT but LDFRT significantly increased the chemosensitivity of both cell lines. This suggests that this new paradigm of mDCF combined to LDFRT could potentially target both cancer and CSCs. However, additional studies, including potential impact on the CSCs niches, are necessary to fully assess the potential efficacy of this approach in these cells.

## 4. Discussion

The traditional role of high-dose fractionated radiation therapy is to achieve local tumor control by inflicting DNA damage to the primary tumor. On the other hand, the purpose of LDFRT is to induce effective cell killing through hyper radiation sensitivity (HRS) and potentiates the effects of chemotherapy [46,47]. This approach, which can safely allow irradiation of larger volumes, could be particularly beneficial for gastric cancer that has disseminated into the peritoneum (carcinomatosis). The current National Comprehensive Cancer Network’s recommendation for patients with gastric cancer who have node-positive disease or cT2 or higher tumors is surgery followed by adjuvant chemoradiation or perioperative chemotherapy. However, significant toxicity has been associated with adjuvant chemoradiation, including 41% of patients developing grade 3 and 32% developing grade 4 toxicity [48]. Nonetheless, it should be pointed out that delivery of radiotherapy has considerably improved since these initial studies largely due to the advent of highly conformal radiotherapy techniques such as 3-dimensional conformal radiotherapy, image-guided radiotherapy (IGRT), intensity-modulated radiotherapy (IMRT), and volumetric modulated arc therapy (VMAT) that now allow extensive normal tissue sparing. Use of IMRT for preoperative rectal cancer, for example, is associated with lower toxicity [49] and a recent re-examination of the Surveillance, Epidemiology, and End Results (SEER) database suggest that sub-population of colon cancer patients (pT4N2M0) who received adjuvant radiation therapy could have significantly better overall survival and cancer-specific survival [50]. Even so, delivery of conventional radiation doses to most colon and disseminated GI cancers still remains a challenging problem mainly due to the proximity of healthy radiosensitive organs (kidneys, liver, small bowel, lungs, heart, and spinal cord) to the targeted tumors. In an effort to address these limitations for disseminated stomach cancer, we developed a pre-clinical mouse model to evaluate the role of a likely biomarker, DUOX2, on the effectiveness of chemopotentiation by LD-WART.

We had initially reported that human gastric cancer cells are hyper-radiosensitive to radiation doses as low as 0.15 Gy, and that DUOX2, an enzyme involved in the production of hydrogen peroxide, played a critical role in mediating HRS in these cells [18]. The mechanisms underlying hyper-radiosensitivity are rather complex and still not fully understood. They have been associated with intrinsic cellular characteristics, failure to arrest in the early G_2_ phase of the cell cycle and increased apoptosis [51,52]. The initial fraction of low dose radiation is believed to cause cell cycle arrest in the most radiosensitive phase of the cell cycle, G_2_/M, resulting in increased cytotoxicity with successive fractions. However, not all cells lacking the capacity to arrest in G_2_ are hyper-radiosensitive, suggesting that other mechanisms could be involved [53]. It has also been established early on that in contrast to high dose radiation, Low-Dose (<0.2 Gy) HRS was not associated with the formation or the persistence of γH2AX foci [54]. This is in good agreement with our study in stomach cancer cells, indicating that our combined regimen of LDFRT and mDCF did not upregulate the DNA damage response in these cells [18]. Our data, however, indicated that the capacity to upregulate DUOX2 and consequently increase ROS was critical to mediate HRS in these cells [18]. This is particularly important since our IHC data indicate that over 30% of stomach adenocarcinoma (STAD) are negative for DUOX2 and an analysis of the TCGA database indicates that DUOX2 mRNA is not upregulated in STAD (Figure 5 and Figure 6). The capacity to upregulate DUOX2 at the protein level in a particular tumor could thus serve as a potential valuable biomarker for chemopotentiation by LD-WART.

Although ROS are pro-tumorigenic, they are also toxic above a certain threshold, and this could be exploited for therapeutic gain. Since normal cells have lower basal ROS levels than cancer cells, they possess a robust capacity to tolerate a sizeable fluctuation in ROS levels [20]. Cancer cells, on the other hand, maintain high basal ROS levels due to enhanced metabolic activity and consequently have a much narrower window of tolerance. This vulnerability of cancer cells implies that subtle and transient burst of ROS could selectively kill cancer cells while sparing normal cells. In fact, this seems to be the case since our data indicate that one cycle of LD-WART combined with mDCF was well tolerated and prevented cancer progression in a human gastric cancer xenograft mouse model expressing DUOX2 (Figure S3 and Figure 2C). In addition, using serum protein oxidation as a read-out of DUOX2 activity, our data indicated that the combined regimen of LD-WART and mDCF resulted in increased serum protein oxidation in DUOX2 positive but not negative tumors (Figure 3). The inability to upregulate ROS combined to HIF-1 high basal level in the DUOX2 negative tumors may have also contributed to their lack of response to LD-WART (Figure 4C and Figure 2D). Although HIF-1 is a well-established mediator of radio-resistance [55], radiation-induced HIF-1 also coincides with tumor reoxygenation [56]. In fact, our data indicate that our combined regimen significantly upregulated several pro-angiogenic factors in the DUOX2 positive but not negative tumors (Figure 4). It thus seems likely that angiogenesis could have contributed to tissue reoxygenation and consequentially radiosensitivity in the DUOX2 positive tumors. There is, however, a complex interplay between ROS, hypoxia, and angiogenesis. While ROS are both necessary and sufficient for HIF-1 activation [57,58], there are also ROS-independent mechanisms that can stimulate HIF-1 through reoxygenation [56]. While our combined regimen did not increase angiogenesis factors in the DUOX2 negative tumors (Figure 4B), it is important to note that their basal levels were higher than in DUOX2 positive tumors. The increased levels of angiogenesis could thus have contributed to increased HIF-1 basal levels in the DUOX2 negative tumors and increased drug uptake in both tumor types, especially since cisplatin can be retained in red blood cells for up to two years after treatment [59]. This interplay between ROS, HIF-1, and angiogenesis may thus be responsible for DUOX2 negative tumors response to chemotherapy and DUOX2 positive tumors response to chemopotentiation by LD-WART (Figure 2).

The mechanisms that lead to DUOX2 upregulation in gastric cancer cells probably involve NF-κB and Sta-1 (Figure 7 and Figure 8). There is considerable cross-talk between ROS and NF-kB signaling. On the one hand, ROS may regulate NF-κB activation to express antioxidant genes and on the other one, activated NF-κB can regulate Nox enzymes, resulting in elevated production of ROS [45]. It thus seems likely that a positive feedback loop occurs where production of H_2_O_2_ in response to DUOX2 upregulation ([18], Figure 1A) results in NF-κB activation, which in turn upregulates DUOX2 (Figure 7). The end product probably relies on whether or not a threshold for ROS tolerance has been exceeded. In the case of stomach cancer cells, it appears that DUOX2 upregulation at the mRNA and protein levels by NF-kB and Stat-1 is sufficient to bypass the tolerance levels ([18], Figure 1, Figure 7 and Figure 8). Because the rapid generation of ROS can also induce CSC death [45,60], we also initiated a pilot study to evaluate whether our approach could be effective in these cells. CSCs are notoriously resistant to conventional therapies, including radiation therapies, and new cancer therapies should strive at targeting both cancer and CSC [43,61]. Higher concentrations of ROS scavengers and neutralization of radiation-induced ROS are contributing factors to CSCs radio-resistance. Our pilot study on CSCs indicates that the combined regimen of mDCF and LDFRT was actually effective in both cancer and CSC-like cells (Figure 9). Given that LDFRT upregulates DUOX2 in these cells (Figure 9A) it seems probable that CSC could also be vulnerable to a sudden rapid burst of ROS that would tip the balance beyond the antioxidant capacity of the cells [20]. However, additional studies, including the potential impact on the CSCs niches, are necessary to fully assess the potential efficacy of this approach in these cells.

While the present study contributes to improving our current understanding of chemopotentiation by LDFRT, it still carries a number of limitations. The use of fluorescence imaging for monitoring cancer cells progression in vivo allowed measurement of cancer progression in real-time but required a large number of cells (28 × 10^6^) per animal to initiate the study. This complicated comparison to our initial cell-based assays that exposed between 300–1000 cells to different modalities [18]. Nonetheless, the in vivo data obtained were consistent with the in vitro data but much less pronounced. One possible way to improve treatment efficiency in vivo would be to repeat the cycle two or three times. This seems reasonable given the low toxicity of these very mild treatments, consisting of mDCF used at 50% of the Maximum Tolerated Dose (MTD) [26] and radiation therapy used at 0.15 Gy per fraction that did not result in any significant toxicity in any treatment groups as evidenced by no body weight loss during the study (Figure S3). Another limitation is the small number of patients’ samples used in the tissue microarray analysis that, while informative, should be correlated with patients’ response to radiotherapy. It also remains to be determined whether this approach could impact local tumor control and to what extent CSCs could be affected.

## 5. Conclusions

This pre-clinical study indicates that one cycle of mDCF used at 50% MTD combined to LD-WART is well tolerated in mice and suggests that DUOX2 could be used as a biomarker for chemopotentiation by LD-WART. The data indicate that tumors positive for DUOX2 are more responsive to the combined regimen of mDCF and LD-WART while DUOX2 negative tumors are more sensitive to chemotherapy alone. This could be important in the event that such an approach reaches clinical applications, given that more than 30% of human stomach adenocarcinoma do not express DUOX2 at the protein level. More studies are needed to further validate this potential biomarker in clinical settings.

## Supplementary Materials

The following are available online at https://www.mdpi.com/article/10.3390/cancers13164186/s1, Figure S1. In vivo imaging of gastric cancer cells. Xenogen imaging of total fluorescent signal after intraperitoneal (ip) injection of 10 and 30 million human gastric cancer cells NCI-N87 stably expressing GFP. The quantitative representation of total fluorescent intensity in the abdomen (mean ± SEM). Xenogen fluorescent filters used: excitation (465 nm) and emission (520–580 nm); exposure time = 1 s. Images were corrected for tissue auto-fluorescence. Figure S2. Schematic representation of the mice treatment plan; Figure S3. Weight measurements of mice bearing NCI-N87 tumors expressing endogenous (A) or reduced (B) DUOX2 and treated with the indicated treatment. Results are expressed as a percentage of changes during the treatment course. *n* = 6–8 mice per group; Figure S4. (A) Mouse angiogenesis profiler array performed on tumors expressing endogenous DUOX2 levels. Three to four tumors of each indicated treatment group were pooled and analyzed as described in the Material and Methods. Data are presented as fold induction of respective control (Vehicle) after background subtraction ± SD. (B) Same as (A), except that proteins were extracted from DUOX2 negative tumors (shDUOX2). Statistical analyses were performed by one-way analysis of variance (ANOVA) using GraphPad Prism 6.0 software. * *p* < 0.05, ** *p* < 0.01, *** *p* < 0.001. *p* < 0.05 was considered significant.
