# Supplementary material for: DUOX2, a New Biomarker for Disseminated Gastric Cancer’s Response to Low Dose Radiation in Mice
Source: Cancers (Basel). 2021 Aug 20;13(16):4186. doi: 10.3390/cancers13164186 (PMC8392330; doi:10.3390/cancers13164186)
Supplement: Supplementary file 1 [file cancers-13-04186-s001.zip › cancers-1342113-supplementary.pdf]

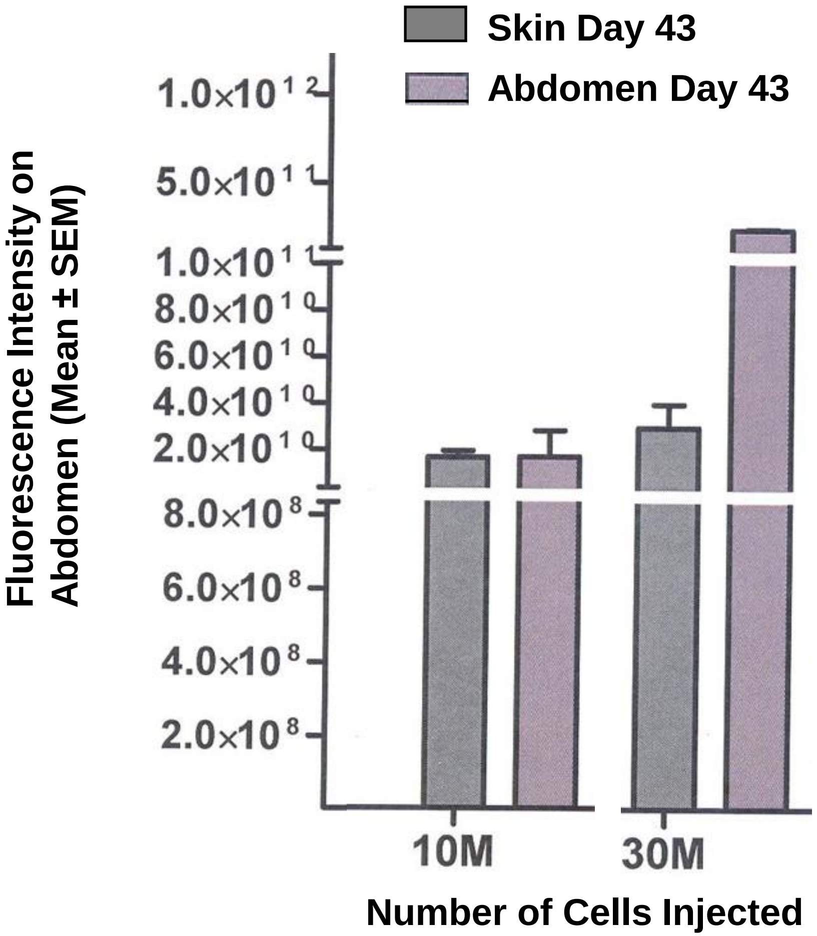

Parekh et al. Figure S1

Mouse treatments

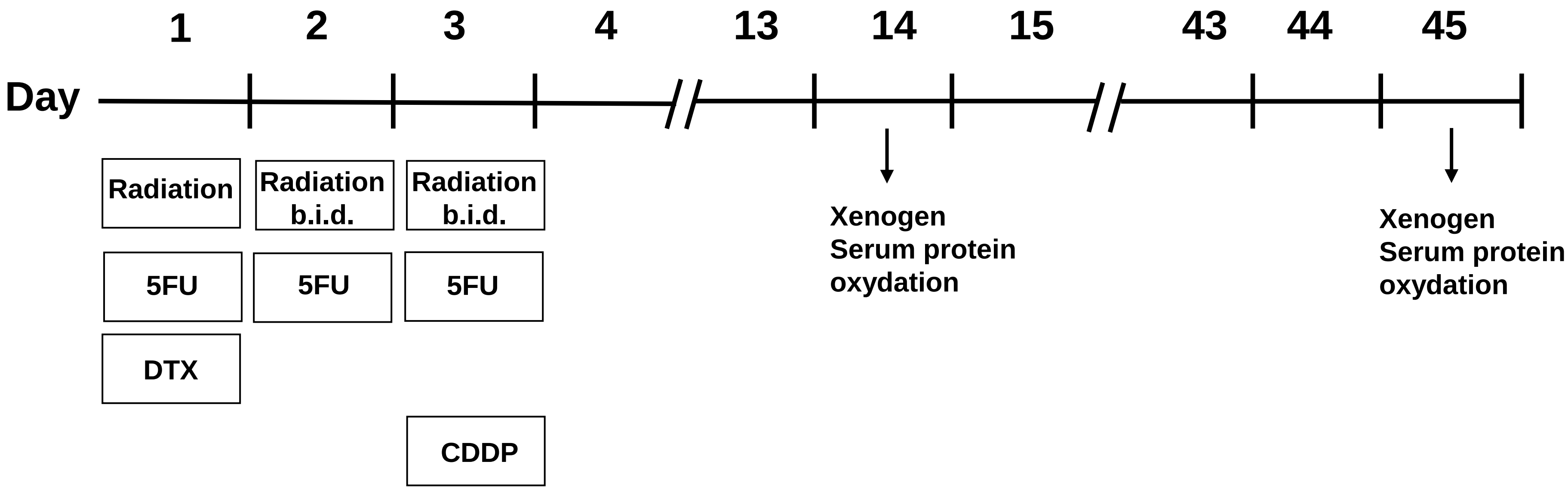

Parekh et al. Figure S2

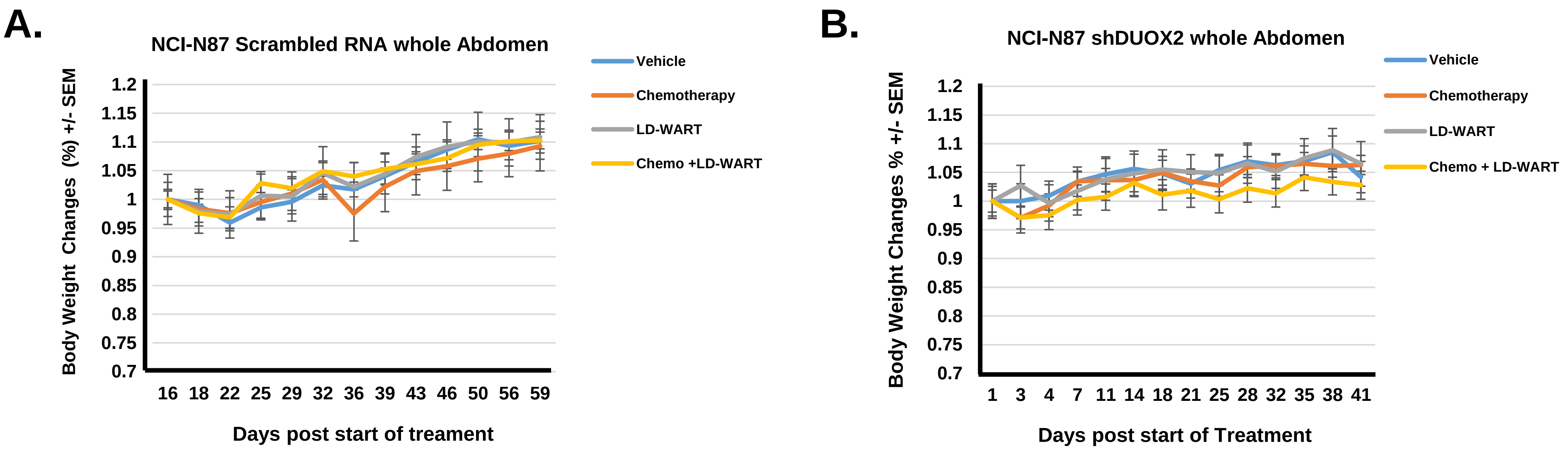

Parekh et al. Figure S3

A.

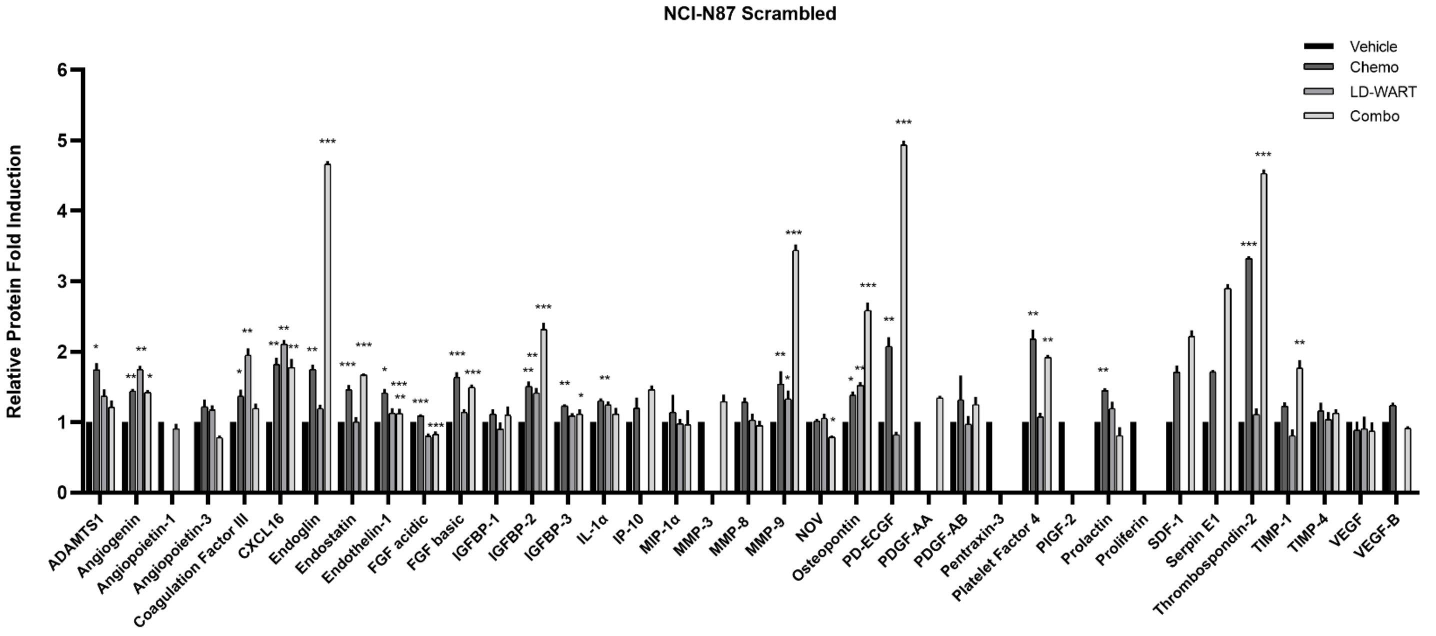

B.

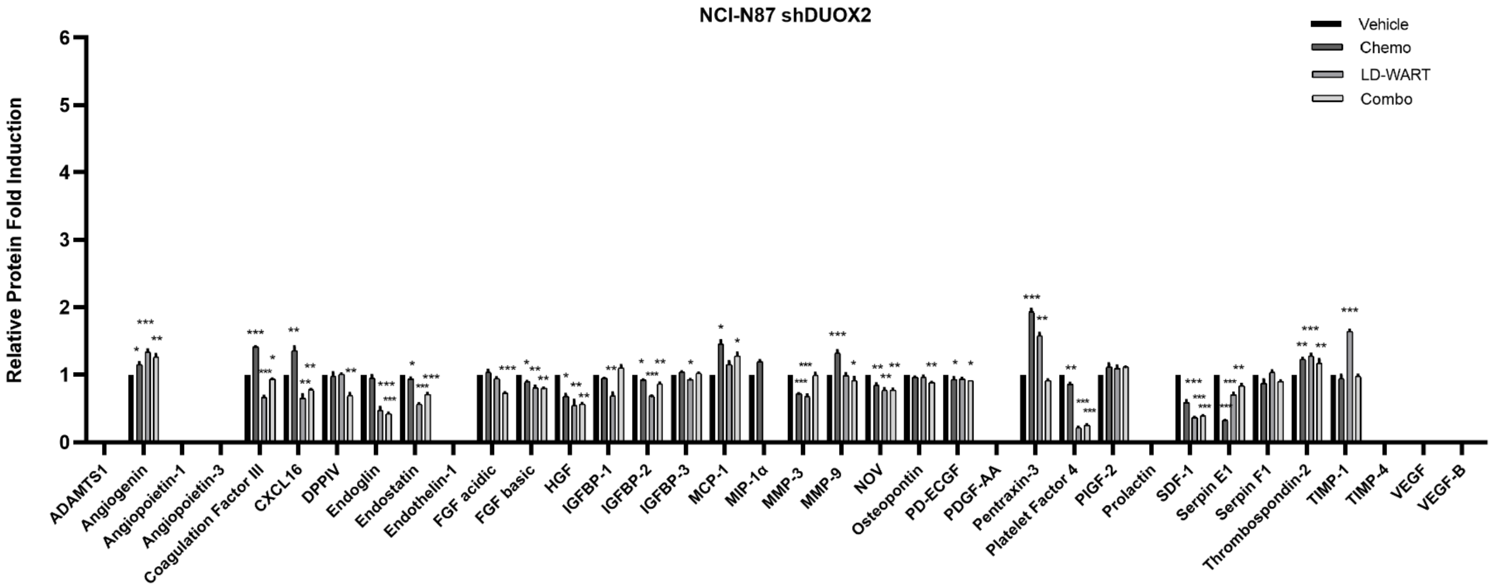

*$p<0.05$
**$p<0.01$
***$p<0.001$

Parekh et al Figure S4

Supplemental Table S1. DUOX2 Negative. Patient Characteristics:

Array position	Sex	Age	Anatomic site	Pathology	Nature	Grade	Stage (TNM)	Intensity in tumor	
								DUOX2	CD68
C01	M	55	Stomach	Adenocarcinoma	Malignant	I	T3N0M0	0	1.5
C03	M	65	Stomach	Adenocarcinoma	Malignant	I~II	T3N1M0	0	3.3
D01	M	55	Stomach	Adenocarcinoma	Malignant	I	T3N0M0	0	1.5
D03	M	65	Stomach	Adenocarcinoma	Malignant	I~II	T3N1M0	0	3.3
E01	M	43	Stomach	Adenocarcinoma	Malignant	II~III	T3N1M0	0	1.5
E02	M	58	Stomach	Adenocarcinoma	Malignant	II~III	T3N0M0	0	NMc
E04	F	51	Stomach	Adenocarcinoma	Malignant	II~III	T3N1M0	0	1
E10	F	45	Stomach	Adenocarcinoma	Malignant	III	T3N0M0	0	3
E11	F	59	Stomach	Adenocarcinoma	Malignant	III	T3N2M0	0	3.1
F01	M	43	Stomach	Adenocarcinoma	Malignant	II~III	T3N1M0	0	1.5
F02	M	58	Stomach	Adenocarcinoma	Malignant	II~III	T3N0M0	0	NMc
F04	F	51	Stomach	Adenocarcinoma	Malignant	II~III	T3N1M0	0	1
F11	F	59	Stomach	Adenocarcinoma	Malignant	III	T3N2M0	0	3.1
G01	M	39	Stomach	Adenocarcinoma	Malignant	III	T2N1M0	0	1.5
G02	M	65	Stomach	Adenocarcinoma	Malignant	III~IV	T3N1M0	0	2
H01	M	39	Stomach	Adenocarcinoma	Malignant	III	T2N1M0	0	1.5
H02	M	65	Stomach	Adenocarcinoma	Malignant	III~IV	T3N1M0	0	2

NMc= No Macrophages cells

Supplemental Table S2. DUOX2 Strong Positive (Intensity ≥ 2.8). Patient Characteristics:

Array position	Sex	Age	Anatomic site	Pathology	Nature	Grade	Stage (TNM)	Intensity in tumor	
								DUOX2	CD68
B12	M	66	Stomach	Adenocarcinoma	Malignant	I	T3N1M0	3.4	3.3
C02	M	52	Stomach	Adenocarcinoma	Malignant	I~II	T2N1M0	3.1	2.5
C12	M	76	Stomach	Adenocarcinoma	Malignant	II~III	T3N0M0	3.4	3.1
D02	M	52	Stomach	Adenocarcinoma	Malignant	I~II	T2N1M0	3.1	2.5
D12	M	76	Stomach	Adenocarcinoma	Malignant	II~III	T3N0M0	3.4	3.1
E03	F	52	Stomach	Adenocarcinoma	Malignant	II~III	T3N1M0	3.3	3.5
E05	M	60	Stomach	Adenocarcinoma	Malignant	III	T2N1M0	2.8	3.1
E07	M	61	Stomach	Adenocarcinoma	Malignant	III	T3N2M0	3	3.5
F03	F	52	Stomach	Adenocarcinoma	Malignant	II~III	T3N1M0	3.3	3.5
F05	M	60	Stomach	Adenocarcinoma	Malignant	III	T2N1M0	2.8	3.1
F07	M	61	Stomach	Adenocarcinoma	Malignant	III	T3N2M0	3	3.5
